# Design and Implementation of the Surveys of Women: Protocol for an Address-Based Sampling Multimodal Study

**DOI:** 10.2196/40675

**Published:** 2023-03-15

**Authors:** Stephanie Poland, Michael Stern, Ned English, Steven Pedlow, Katherine Archambeau, Kari Carris

**Affiliations:** 1 National Opinion Research Center at the University of Chicago Chicago, IL United States; 2 Department of Media & Information College of Communication Arts and Sciences Michigan State University East Lansing, MI United States

**Keywords:** multimode, web survey, cross-sectional, women’s health, reproductive health, methods, data collection, implementation, survey, contraceptive health, contraception, survey methods, data collection, data processing

## Abstract

**Background:**

Studies conducted in the United States such as the National Survey of Family Growth (NSFG) and the Pregnancy Risk Assessment Monitoring System (PRAMS) collect data on pregnancy intentions to aid in improving health education, services, and programs. PRAMS collects data from specific sites, and NSFG is a national household-based survey. Like NSFG, the Surveys of Women was designed to survey participants residing in households using an address-based sample and a multimode data collection approach. The Surveys of Women collects data from eligible participants in 9 states within the United States on contraception use, reproductive health, and pregnancy intentions. In this paper, we focus on the baseline data collection protocol, including sample design, data collection procedures, and data processing. We also include a brief discussion on the follow-up and endline survey methodologies. Our goal is to inform other researchers on methods to consider when fielding a household-level reproductive health survey.

**Objective:**

The Surveys of Women was developed to support state-specific research and evaluation projects, with an overall goal of understanding contraceptive health practices among women aged 18-44 years. The project collects data from respondents in 9 different states (Arizona, Alabama, Delaware, Iowa, Maryland, New Jersey, Ohio, South Carolina, and Wisconsin) over multiple rounds.

**Methods:**

Households were selected at random using address-based sampling methods. This project includes a cross-sectional baseline survey, 2 or 3 follow-up surveys with an opt-in panel of respondents, and a cross-sectional endline survey. Each round of data collection uses a multimode design through the use of a programmed web survey and a formatted hard copy questionnaire. Participants from the randomly selected households access their personalized surveys through a web survey or mail in a hard copy questionnaire. To maximize responses, these surveys follow a rigorous schedule of various prompts bolstering the survey implementation design, and the participants received a modest monetary incentive.

**Results:**

This is an ongoing project with results published separately by the evaluation teams involved with data analysis.

**Conclusions:**

The methods used in the first baseline survey informed modifications to the methods used in subsequent statewide surveys. Data collected from this project will provide insight into women’s reproductive health, contraceptive use, and abortion attitudes in the 9 selected states. The long-term goal of the project is to use a data collection methodology that collects data from a representative sample of participants to assess changes in reproductive health behaviors over time.

**International Registered Report Identifier (IRRID):**

DERR1-10.2196/40675

## Introduction

National studies, such as the National Survey of Family Growth (NSFG) and the Pregnancy Risk Assessment Monitoring System (PRAMS), collect data on pregnancy intentions among women of childbearing age to aid in improving health education, services, and programs. Unintended pregnancies, in particular, can have negative health, economic, and psychological impacts on individuals and families. While the rates of unintended pregnancy have declined nationally in the United States since 2008, data indicate that 45% of pregnancies in 2011 were unintended [1,2]. At the state level, the rates of unintended pregnancy among women aged 15-44 years vary from 32 per 1000 women to 62 per 1000 women, with Delaware reporting the highest rate [3].

To support state-specific research and evaluation projects, the Surveys of Women was developed to assess changes in pregnancy intentions and attitudes toward contraception. Initially conceptualized as 2 cross-sectional surveys (a baseline survey followed by an endline survey approximately 5 years later), the project evolved to include follow-up surveys with an opt-in panel to meet the needs of the evaluation team. The Surveys of Women was first launched in the states of Delaware and Maryland with the administration of the baseline survey in 2016.

Data collection for this study used an address-based sampling (ABS) multimode approach. ABS methodological approaches have become the preferred method for respondent selection, compared with random digit dialing designs that rely on telephone interviews. Randomly selecting addresses from a list of known households in an area allows researchers to maximize coverage and improve response rates [4]. Further, the data collection methodology used a series of mailings and nonresponse follow-up activities as recommended by Dillman et al [5,6]. The Dillman method is a well-established method that recommends using distinct contacting strategies to encourage response.

The project increased in scope to support additional research, which included the incorporation of additional evaluation teams as the project expanded into new states and the addition of a follow-up component between the baseline and endline surveys. In addition to Delaware and Maryland, the project has conducted surveys in Arizona, Alabama, Iowa, New Jersey, Ohio, South Carolina, and Wisconsin.

The following report summarizes the study methodology used for the baseline surveys in each state, with a section on the planned differences for the follow-up survey administration.

## Methods

### Study Overview

The Surveys of Women are multimode, multistate studies with the overarching aim of measuring contraceptive use and understanding reproductive health practices among women aged 18-44 years in 9 states. Data are collected over multiple rounds—a baseline cross-sectional survey, 2 to 3 follow-up surveys, and an endline cross-sectional survey. During the baseline survey, households were selected at random using ABS methods, with any 18- to 44-year-old woman in the household eligible to participate. Women from the baseline could opt-in to participate in additional follow-up surveys. The final stage is a second cross-sectional endline survey. For the purpose of this paper, we will address the methodology for the baseline surveys followed by a brief discussion of the follow-up survey methodology. We also briefly discuss the endline survey, which will follow the same methodology as the baseline administration.

### Ethical Considerations

All questionnaires and data collection methods were reviewed and approved by the NORC (National Opinion Research Center at the University of Chicago) institutional review board (IRB). NORC's IRB has obtained a Federal Wide Assurance and is registered with the US Federal Office for Human Research Protections (FWA #00000142). The IRB protocol submitted included sufficient detail on the methodology to ensure that subjects’ privacy is protected, and data confidentiality is maintained. Materials reviewed for the Surveys of Women included a description of the study’s sample, the methodology used including recruitment methods and incentives, the informed consent language, and the survey questionnaire. All survey administrations (baseline, follow-ups, and endline) included informed consent information for participants on the login page for the web survey and the first page of the formatted hard copy. The consent information included standard provisions related to the voluntary nature of the survey, the right to refuse to answer any question, the confidential nature of responses, the incentives provided, and the contact number for the IRB representative in the event of questions about their rights as a study participant. A specific project toll-free number and email address were provided to respondents as well as for questions and concerns.

### Sample Design

#### Overview

The target number of completed surveys for each baseline was 2000 per state; therefore, the sampling plan for each baseline survey was designed to meet these targets. The sample frame for the cross-sectional baseline surveys was the United States Postal Service computerized delivery sequence (CDS) file enhanced with age-targeted lists [7,8]. The CDS was licensed from an independent vendor and is also referred to as the DSF (delivery sequence file). We geocoded the CDS and, using the geocodes, appended the file with area-level demographic information from the American Community Survey for purposes of stratification. To maintain the coverage of the CDS and allow for oversampling of women with a particular characteristic, the updated CDS frame was deduplicated against a list procured from a vendor—a sample of addresses likely to contain a woman in the target age range of 18-44 years.

#### Stratification

In all states, the sample was stratified into “high” and “low” density areas for specified variables of interest, such as minority status, rurality, or poverty level. The variables of interest and geographic unit varied depending on the state as shown in Table 1. We adjusted the degree of stratification between the baseline in the original sites (Delaware, Maryland, South Carolina, and Alabama) and subsequent sites that targeted minorities in order to increase the share of non-White, non-Hispanic respondents. This approach resulted in 4 strata per state: CDS-only low density, CDS-only high density, list low density, and list high density, where list refers to those likely to contain women in the target age range. The CDS-only high-density and list high-density strata were oversampled relative to the respective low strata in each baseline data collection.

**Table 1:** Baseline sample size and variables of interest.

State	Variables of interest	Unit of stratification	Overall addresses released, n	CDS^a^-only low density, n	CDS-only high density, n	List low density, n	List high density, n
Delaware	Non-White, non-Hispanic population; poverty status	Census tract	14,375	3229	5417	1875	3854
Maryland	Non-White, non-Hispanic population; poverty status	Census tract	13,875	2817	5425	1982	3651
South Carolina	Rural populations (in low-density counties)	County	18,400	6600	4000	4900	2900
Alabama	Rural populations (in low-density counties)	County	18,400	5200	4800	4200	4200
Iowa	Non-White, non-Hispanic population; poverty status	Census tract	14,700	5400	3200	4300	1800
Ohio	Rural Appalachia	County	20,100	9300	2700	6400	1700
Arizona	Non-White, non-Hispanic population; poverty status	Census tract	18,138	7885	1559	6505	2189
New Jersey	Non-White, non-Hispanic population; poverty status	Census tract	24,499	9531	3767	8518	2683
Wisconsin	Non-White, non-Hispanic population; poverty status	Census tract	12,481	4886	1679	4264	1652

^a^CDS: computerized delivery sequence.

### Questionnaire

#### Overview

The goal of each questionnaire was to capture data on respondents’ contraceptive use, pregnancy history, health status, and opinions on abortion. Variations among individual state questionnaires existed, including items tailored to their respective state, such as insurance plans or state-specific clinics. All questionnaires were offered in English and Spanish languages.

#### Questionnaire Development

The initial questionnaire (developed for the Delaware and Maryland baseline survey) consisted of items drawn from existing sources, such as the American Community Survey, the Behavioral Risk Factor Surveillance System, the NSFG, the PRAMS, and the Delaware Household Survey. A copy of the questionnaire can be found in Multimedia Appendix 1.

Subsequent baseline state questionnaires were created using the original questionnaire developed for Delaware and Maryland. Prior to the start of each baseline survey, the respective evaluation teams had the option to propose new questions relevant to their goals or propose modifications to existing items.

All questionnaire items were formatted to be self-administered and were formatted with the goal of visual consistency and presentation among self-administered modes (ie, web and paper self-administered questionnaire [SAQ]). For example, questions that were programmed as grids on the web were formatted as such on the paper version. Similarly, items where a follow-up question was embedded into the stem question were indented with arrows directing the respondent to the correct follow-up item on the paper version. A computer-assisted telephone interview (CATI) was programmed and administered during the Delaware, Maryland, South Carolina, and Alabama baseline surveys. Due to low productivity rates with the CATI survey (ie, only 9 completed CATI interviews), the telephone survey was abandoned after the South Carolina and Alabama baseline surveys.

#### Cognitive Interviews

A total of 11 cognitive interviews (8 with English speakers and 3 with Spanish speakers) were conducted prior to the start of data collection in Delaware and Maryland. Cognitive interviews allow researchers to identify any potential issues within the questionnaire, such as question comprehension, illogical flow, and appropriateness of response options. As a result of cognitive interviews, duplicative questions were identified and removed, double-barreled questions were corrected, response options were modified, and images for birth control methods (eg, intrauterine device, implant, and birth control pills) were added to clarify for respondents what each type of birth control was.

In addition, cognitive interviews were conducted with 10 South Carolina residents and 10 Alabama residents prior to the data collection launch. Cognitive interviews were not conducted prior to the launch of data collection in Iowa, Ohio, Arizona, New Jersey, or Wisconsin as these surveys used established items that did not require additional review.

### Survey Design

**Figure 1:**
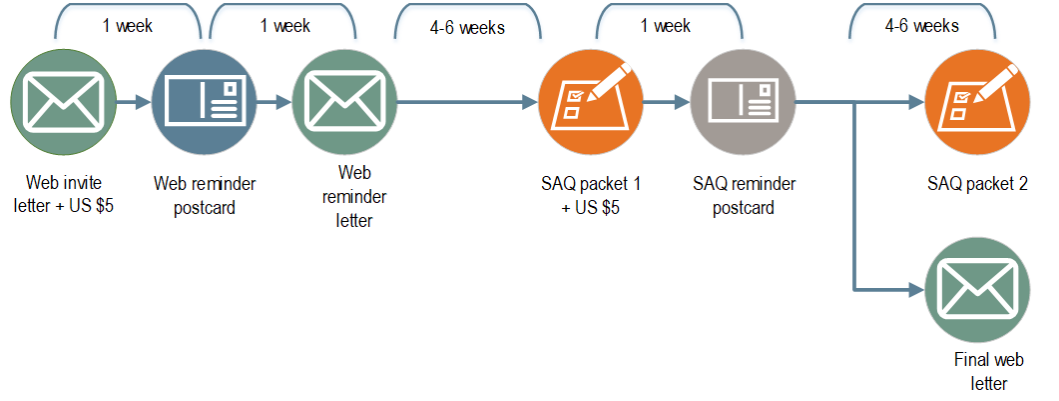
Baseline survey case flow. SAQ: self-administered questionnaire.

All Surveys of Women data collections follow a similar methodology, with a series of prompts to encourage response as shown in Figure 1. Respondents were offered the option to complete the web survey first; those who do not complete via the web survey were sent a paper SAQ. A vendor-supplied flag appended to the sample file indicated which addresses may have a Spanish speaker and would benefit from bilingual materials.

### Data Collection Methods

#### Respondent Mailings or Prompts

A Surveys of Women study logo was developed and displayed on all printed materials and within the header of the web survey. Four images that represented each state were selected and displayed as an image carousel on the state’s web survey login page as well as static images on the front cover of the state’s paper questionnaire. All respondent materials contained a project-specific email address and toll-free telephone number should respondents have a question about the study or their participation.

The baseline surveys in each state followed a similar protocol when mailing printed copies of materials. The baseline surveys for Delaware, Maryland, South Carolina, and Alabama offered CATI as a third mode of data collection. A subset of nonrespondents whose addresses could be matched to a telephone number were contacted by trained telephone interviewers to complete the survey over the telephone. Due to low overall response in these 4 states, it was deemed that CATI was not a viable option for attaining completion for this population and was dropped from subsequent survey administrations.

Each mailing was sent to the sampled address and addressed to “[STATE] Resident.” The types of respondent contacts are described below.

#### Web Mailings

##### First Web Invitation Letter

The first point of contact was an invitation letter, which described the purpose of the study and provided the survey link and the respondents’ unique personal identification number. Included with the letter were a noncontingent cash incentive (a US $2 bill for Delaware and Maryland residents and a US $5 bill for all other survey administrations) and a web instructional insert, which showed how to access the survey.

##### Web Reminder Postcard

Approximately 1 week after the web invitation letter, a reminder postcard was sent to all sampled households reminding them to participate.

##### Second Web Letter

Sampled addresses that had not responded to the first 2 mailings were sent a second letter requesting participation via the web survey instrument.

##### Last Chance Letter

A final letter was sent to those who had not yet responded during the final weeks of data collection. The purpose of the letter was to encourage participation before data collection closed.

#### SAQ Mailings

##### SAQ Packet 1

Paper questionnaires were sent to sampled addresses who had not responded to the web survey invitation. The packets contained a cover letter, a copy of the questionnaire, an additional noncontingent cash incentive (a US $1 bill for Delaware and Maryland and a US $5 bill for other states), and a postage-paid envelope to return the completed questionnaire.

##### SAQ Reminder Postcard

Approximately 1 week after sending the initial paper questionnaire packet, a reminder postcard was sent to sampled addresses to remind them to complete and return their paper questionnaire.

##### SAQ Packet 2

A second and final paper questionnaire was sent to sampled addresses who had not completed the web survey or returned a paper version of the questionnaire. The second packet contained a cover letter, paper questionnaire, and postage-paid return envelope.

#### Household Rostering

With almost 10% of households estimated to contain more than 1 eligible respondent, it was important to collect data from additional eligible women in the household, particularly as their reproductive health choices may be different. Therefore, during the baseline surveys, respondents had the option to provide the name and email address of up to 2 additional women, aged 18-44 years, who were living in the household. If additional women were rostered through the web survey, we automatically sent them an invitation letter and unique personal identification number at the email address specified. Women who were rostered via the paper questionnaire were sent an email invitation once the data from the questionnaire had been entered, and the name and email address were made available to project staff.

#### Incentives

##### Noncontingent Cash Incentives

All web invitation letters and initial paper questionnaire packets included a noncontingent cash incentive. In the Delaware and Maryland baseline survey, the noncontingent cash incentive was a US $2 bill for the web letter and a US $1 bill for the first paper questionnaire mailing. Due to a lower-than-anticipated response for that baseline study, all future baseline studies increased the preincentive amount to US $5 for both the web invitation letter and the initial paper questionnaire packet mailing.

##### Postsurvey Completion Incentives

Respondents who completed the baseline survey received a US $10 postincentive in the form of an electronic gift code. Initially, the postincentive offered was in the form of an Amazon gift code. Participants in Arizona, New Jersey, and Wisconsin baseline surveys were offered their choice of an Amazon, Target, or Walmart electronic gift code at the completion of the baseline survey.

### Data Processing

#### Eligibility

At the start of the baseline surveys, two screening questions were asked of household members: (1) “What year were you born?” and (2) “What is your gender?” For those who refused to provide their year of birth, a follow-up question was asked with a list of age categories from which the respondent could select the appropriate age range. To note, Delaware and Maryland used a variation of the age screener item at baseline, originally asking “What is your age (in years)?” These screening questions served to confirm eligibility—a woman aged 18-44 years at the sampled address. Those individuals who did not meet the screening criteria were screened out of the survey, and no further data were collected. Those who were eligible continued into the main questionnaire.

Throughout data collection, cases were reviewed for eligibility. The web was programmed with automated screen-out language, and the paper questionnaire was printed with instructions for the respondent to stop responding and return the questionnaire if either screening question made them ineligible. During postprocessing, ineligible respondents who disregarded these instructions and completed the questionnaire were identified. The main questionnaire data for these respondents were not included in the final data set, and the overall case status was changed to an ineligibility status.

#### Weighting

##### Overview

Weights were applied to all data sets and underwent the same set of steps: (1) base sampling weight (*W*_1_); (2) adjustment for unknown eligibility (*W*_2_); (3) adjustment for nonresponse to the questionnaire (*W*_3_); (4) adjustment for household size (*W*_4_*_a_*); and (5) poststratification (*W*_4_).

##### Step 1: Base Sampling Weight

All sample lines received a base weight, which reflects the probability of a household being selected and is equal to the inverse of the probability of selection. Each stratum had a different base weight, with the base weight for the list sample being equal to the inverse of the sum of the list probability and the DSF probability.

Examples of the list strata versus DSF strata base weight calculations are:

Stratum 1 (Delaware low-density DSF)



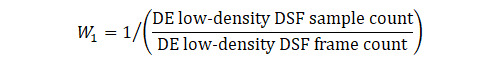



Stratum 2 (Delaware low-density list)



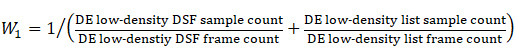



##### Step 2: Adjustment for Unknown Eligibility

This adjustment to the weights accounted for those who were unable to be contacted and whose eligibility status was unknown. This adjustment used the Census’ Public Use Microdata Sample (PUMS) data to estimate household eligibility for each stratum within a state. Completed cases had no adjustment, while incomplete cases received this calculated rate.

##### Step 3: Adjustment for Interview Nonresponse

This weight adjustment compensated for differences in response across eligible survey subgroups. Adjustment cells for the interview nonresponse rate are usually defined by state and high or low density.

##### Step 4: Adjustment for Household Size

The final weight adjustment accounted for within-household eligibility. PUMS data were used to estimate within-household eligibility using race or ethnicity as a comparison point. That is, it was assumed that households with 1 respondent would have the same number of eligible women as average PUMS household data with more than 1 eligible women in the same state and race or ethnic group. The same was true for households with 2 or 3 eligible respondents.

##### Step 5: Poststratification

This step was a 2-part process—imputation first and then raking. Hot-deck imputation was used to impute missing values for the variables used during the raking process. Across the baseline studies, the missing values imputed for the raking variables ranged from approximately 0.6% to approximately 18%.

Once the values had been imputed, raking was performed across 8 variables—age, nativity, employment, marital status, race or ethnicity, housing tenure, children younger than 18 years in the household, and education crossed with income. The purpose of raking was to reduce bias in survey estimates and achieve representativeness of the target population across those variables of interest. The 8 variables selected represent optimal design based on criteria, such as design effect, weight distribution, and the impact of raking on select outcome variables.

### Follow-up Survey Methods

#### Overview

As mentioned previously, the project includes a set of follow-up surveys that occur in the years between the baseline and the endline surveys. The motivation for the follow-up surveys is to continue to assess contraceptive use and changes in attitudes among participants. The sample for each state’s follow-up surveys consists of baseline survey participants who agreed to be recontacted for future studies. Table 2 shows the number of initial respondents who opted into each state’s follow-up survey.

Participants can request to be removed from future contact at any point but remain in the panel. In addition, participants are removed from the study panel if the study team learns that the participant was not actually eligible to complete the baseline survey (eg, older than 44 years or younger than 18 years at the time they completed the baseline survey).

**Table 2:** Panel size by state.

State	Initial panel size, n
Delaware	983
Maryland	957
Alabama	1675
South Carolina	1658
Iowa	1946
Ohio	2066
Arizona	1588
New Jersey	1600
Wisconsin	1577

#### Methodology

##### Overview

All follow-up studies follow a similar data collection methodology when mailing printed copies of materials as described earlier for the baseline survey. However, there are a few deviations from the baseline survey protocol, which include (1) locating current contact information, (2) incorporating email and SMS text message prompts, and (3) offering incentive amounts. These differences are described below.

##### Locating

As the follow-up surveys follow a panel of respondents over a period of up to 3 years, using updated and adequate contact information for the panel is key. Follow-up surveys will be conducted 3 times for participants in South Carolina, Alabama, Iowa, and Ohio, while participants in Delaware, Maryland, Arizona, New Jersey, and Wisconsin will only be contacted for 2 follow-up surveys. Prior to the start of a follow-up survey, the project team sends an email to all panel members who provided an email address with a request to confirm or update their existing contact information.

Throughout data collection, the project team may also conduct individual searches via Accurint, a commercially available locating tool developed by LexisNexis, to locate updated address information for those individuals who have not responded to the mailings or provided the project team with an updated mailing address.

##### Email Prompts

During each baseline survey, participants were asked to provide their name and contact information (address, telephone number, and email address) to facilitate contacting them for future studies. Email addresses are used to send email prompts and serve as another manner of respondent contact. For participants who had an email but not a name, their initial invitation to participate in the follow-up is sent via email.

The number of prompts and timing of these reminders are at the discretion of the data collector, though all follow-up studies conclude data collection with 1 final email prompt to any sample member who has not yet responded.

##### SMS Text Message Prompts

Women who participated in the first follow-up surveys for Iowa and Ohio and the baseline surveys for Arizona, New Jersey, and Wisconsin could consent to future contact via SMS text message. Therefore, SMS text message reminders are used for participants in these states. The SMS text messages are sent with an embedded link to the state’s web survey log-in page and provide respondents with another way to access the survey. SMS text messages were only sent to participants who consented to receive these messages and provided a valid telephone number.

##### Incentives

For those who participate in the follow-up studies, their postincentives are still in the form of electronic gift codes though in increasing amounts. The postincentive for the first follow-up is US $20; it is US $30 for the second follow-up and US $40 for the third follow-up.

#### Eligibility

Eligible respondents who completed the baseline survey and agreed to future contacts constituted the panel of respondents who would be contacted for the state’s follow-up studies. As with all follow-up surveys, it is important that the same respondent participates in each round. As a measure of confirming the individual who responded to a follow-up survey was the same as the original baseline respondent, select demographic variables (age, education, and race) are used as confirmation variables.

During the first follow-up surveys for Delaware, Maryland, South Carolina, and Alabama, these demographic variables were prefilled with the baseline response (if known) into the web survey. The respondents would see the response prefilled and were asked to confirm or update their response.

Regular data reviews, including a review of the demographic variables, are completed during the follow-ups. The study team follows these general steps when reviewing completed cases: (1) Names are compared; if the names match, no further action is taken. (2) If the name does not match, email addresses are reviewed. If email addresses match, no further action is taken. (3) If both the name and email address do not match, responses to age, education, and race are reviewed. If these variables are consistent or there is only 1 variable flagged as a discrepancy, the case is considered a match and no further action is taken. (4) If more than 1 demographic variable is flagged and there is no reasonable explanation for the change (eg, age discrepancy due to change in how the question was asked, increase in education), the case is considered a nonmatch and flagged as the incorrect respondent.

The process to confirm eligibility was refined prior to the start of the first follow-up survey in Iowa and Ohio so that the check on the selected demographic variables was performed systematically. The 5 demographic questions—items about age, gender, ethnicity, race, and education—were moved to the beginning of the survey. Respondents did not see their responses from a prior round; however, the survey was programmed to compare responses to age, education, and race against preloaded values for the case.

With this process, flags are assigned if a response differed from the one previously provided. Respondents continue into the survey if 1 or no flags are triggered. Cases with 2 or more flags indicate discrepant responses; these respondents are taken to a new screen that informs them there is an error with the survey and requests they contact us. Respondents who reach this screen are unable to log back into the survey and are shown this screen until they contact the project team to verify they are the eligible person.

#### Weighting

The follow-up surveys follow the same weighting methodology as described earlier for the baseline surveys.

### Endline Survey Methods

The endline surveys for all states will follow the same sampling approach, data collection methodology, and weighting plan as used for the baseline surveys described earlier. The first endline survey was fielded in 2020-2021 in Delaware and Maryland; at the time of this writing, data processing was underway. Endline survey data collection for the remaining states will continue through August 2023.

## Results

This is an ongoing research project and results from data collected from this study will be published independently by separate research and evaluation teams.

## Discussion

### Conclusions

In summary, the Surveys of Women project follows a standard approach to multimode data collection, which uses a series of prompts to encourage response to the survey. This data collection approach is informed by Dillman et al [5,6] research on multimode surveys and is widely documented in the extant survey methods literature. Additionally, using an address-based sample for the baseline allows the survey to maintain adequate coverage within a state, with the sample stratified into areas in an effort to reach target populations. This project provides an opportunity to use a well-established survey methodology to capture data from a target population on potentially sensitive topics. We will also learn which overall methodology works best for initial participation and subsequent participation in the opt-in panel for follow-up studies, which will be useful for any subsequent, similar projects.

### Limitations

As in any household-based web or mailed questionnaire study, there is no accurate or cost-effective way to confirm that all participants met eligibility criteria definitively. Respondent verification was included in the opt-in follow-up surveys to confirm whether the same individual was participating across rounds, but respondent verification is not feasible for the cross-sectional baseline and endline surveys.

Additionally, as noted in “Data Collection Methods” section, we increased our preincentive amounts and modified our follow-up language after baseline participation, and follow-up panel opt-ins were less than expected in Delaware and Maryland. The changes in approach were applied to the subsequent state surveys and allowed for a more robust sample in those states comparatively.

Another limitation to the survey is that it was offered only in English and Spanish languages. Additionally, as a self-administered survey, it relied on respondent literacy for questionnaire completion and comprehension.

## Appendix

Multimedia Appendix 1 Draft copy of the questionnaire items used in the Delaware and Maryland baseline survey.

## Abbreviations

ABS: address-based sampling
CATI: computer-assisted telephone interview
CDS: computerized delivery sequence
DSF: delivery sequence file
IRB: institutional review board
NSFG: National Survey of Family Growth
PRAMS: Pregnancy Risk Assessment Monitoring System
PUMS: Public Use Microdata Sample
SAQ: self-administered questionnaire

## Data Availability

The data sets generated and analyzed during the Surveys of Women study are not publicly available at this time due to the exclusive data usage rights of the funded research and evaluation teams. Data may be made available from the corresponding author upon reasonable request. Data may also become available as restricted use or public use data sets at some point in the future.
