# Supplementary material for: Design and Implementation of the Surveys of Women: Protocol for an Address-Based Sampling Multimodal Study
Source: JMIR Res Protoc. 2023 Mar 15;12:e40675. doi: 10.2196/40675 (PMC10131823; doi:10.2196/40675)
Supplement: Multimedia Appendix 1 [file resprot_v12i1e40675_app1.docx]

| [STATE] Survey of Women |
| --- |

**SCREENER ITEMS**

1. **[AGE] What year were you born?** [Source: BRFFS]

__ __ __ __ year

1. Prefer not to answer

**[AGE_PROBE]** **What if I give you some age categories?**

1. Under 18
2. 18-19
3. 20-24
4. 25-29
5. 30-34
6. 35-39
7. 40-44
8. 45 or older
9. Don’t know
10. Prefer not to answer
11. **[GENDER] What is your sex?**
12. Female
13. Male
14. Prefer not to answer

**[PROGRAMMR NOTE: Only those who select “female” and those who are between the ages of 18 and 44 advance into the survey. Those that are ineligible should go to OTHWOMEN and then see the ineligible screen.]**

**MAIN QUESTIONNAIRE**

1. **[HEALTH] Would you say that in general your health is…**  [Source: BRFFS]
2. Excellent
3. Very Good
4. Good
5. Fair
6. Poor
7. Prefer not to answer
8. **[HLTHCARE] In the past 12 months, was there any time when you needed healthcare for yourself, for any reason but didn’t get it?** [Source: Modified from University of Massachusetts Medical School. Office of Survey Research. Connecticut Health Care Survey, 2012-2013. Ann Arbor, MI: Inter-university Consortium for Political and Social Research [distributor], 2014-11-05. https://doi.org/10.3886/ICPSR35475.v1]
9. Yes
10. No **🡪 GO TO [BIRTHCTL_EVER]**
11. Prefer not to answer **🡪 GO TO BIRTHCTL_EVER**
12. **[NOHLTHCARE] Why didn’t you get health care for yourself?** *Please check all that apply.*
13. I couldn’t afford it.
14. I did not know where to go.
15. It was too far away.
16. I could not get there when it was open.
17. I could not get an appointment soon enough.
18. I did not have transportation.
19. I didn’t have time to go.
20. I was worried that it wasn’t covered under my insurance.
21. Some other reason why

*Please specify why:* __________________________________________

1. Prefer not to answer

**The next few questions are about using birth control. Birth control refers to ALL the different methods for preventing pregnancy, including condoms, birth control pills, Depo-Provera (the shot), IUDs, implants, and non-prescription methods like withdrawal or “pulling out.” It also includes permanent methods like sterilization (tubes tied or vasectomy) and other methods you might use, even if you used them for reasons other than to prevent pregnancy.**

1. **Have you ever used any of these birth control methods, even if you used the method only once?** *Please check all that apply.* [Source: Modified from NSFG]
2. **[BIRTHCTL_A] Withdrawal (also called “the pull-out method’)**
3. Yes
4. No
5. Don’t know
6. Prefer not to answer
7. **[BIRTHCTL_B] Birth control pills**
8. Yes
9. No
10. Don’t know
11. Prefer not to answer
12. **[BIRTHCTL_C] Birth control patch (Evra® or other)**
13. Yes
14. No
15. Don’t know
16. Prefer not to answer
17. **[BIRTHCTL_D] Vaginal ring (Nuvaring® or other)**
18. Yes
19. No
20. Don’t know
21. Prefer not to answer
22. **[BIRTHCTL_E] Depo-Provera® (also called “the shot”)**
23. Yes
24. No
25. Don’t know
26. Prefer not to answer
27. **[BIRTHCTL_F] IUD (Mirena®, Paragard®, Skyla®, Liletta®, or Kyleena**®**)** *An IUD (IUD stands for “intra-uterine device”) is a birth control device that is inserted inside a woman’s uterus. Some IUDs such as Mirena®, Liletta®, and Skyla® contain a small amount of hormones which are released slowly over many years (3-5 years depending on the IUD) and prevent a woman from getting pregnant. The Paragard® IUD is made of copper, it doesn’t contain any hormones, and can prevent a woman from getting pregnant for up to 10 years.*
28. Yes

**[IUD_EVER]** **What IUD have you used?** *Please check all that apply.*

- 1. Liletta® (hormonal IUD used for up to 3 years)
  2. Mirena® (hormonal IUD used for up to 5 years)
  3. Skyla® (hormonal IUD used for up to 3 years)
  4. Paragard® (non-hormonal IUD used for up to 10 years, also called the “copper IUD”)
  5. Kyleena® (hormonal IUD used for up to 5 years)

1. Don’t know
2. Prefer not to answer

**[IUDTRBLEVER] Did you ever have any trouble with the IUD once it was inserted? For example, did it ever fall out?**

- - 1. Yes 🡪 *Please specify difficulty:* ______________________________________
    2. No

1. Prefer not to answer

2. No

**[NOIUDUSE] What is the primary reason you have never used an IUD?**

1. The cost is too high
2. I am not familiar with this birth control method
3. Due to my beliefs (religious or otherwise)
4. To avoid negative side effects
5. I am not comfortable requesting an IUD from my doctor
6. I am concerned about the procedure for inserting or removing the IUD
7. My provider did not discuss IUDs with me
8. I prefer to use a different method
9. I don’t want an object in my body
10. Other, *please specify:* ____________________________________________
11. Don’t Know
12. Prefer not to answer
13. **[BIRTHCTL_G] Implant (Implanon® or Nexplanon®)** *The birth control implant is called Nexplanon® (the older version was called Implanon®). The implant is a single rod that is inserted inside a woman’s arm. The implant contains a small amount of hormones that are slowly released to prevent a woman from getting pregnant. The implant is effective for up to 3 years.*
14. Yes
15. No

**[NOIMPLANT] What is the primary reason you have never used an implant?**

1. The cost is too high
2. I am not familiar with this birth control method
3. Due to my beliefs (religious or otherwise)
4. To avoid negative side effects
5. I am not comfortable requesting an implant from my doctor
6. I am concerned about the procedure for inserting or removing the implant
7. My provider did not discuss implants with me
8. I prefer to use a different method
9. I don’t want an object in my body
10. Other, *please specify:* ________________________________________
11. Don’t know
12. Prefer not to answer
13. **[BIRTHCTL_H] Male condoms**
14. Yes
15. No
16. Don’t know
17. Prefer not to answer
18. **[BIRTHCTL_I] Barrier methods (diaphragm, sponge, cervical cap, female condom)**
19. Yes
20. No
21. Don’t know
22. Prefer not to answer
23. **[BIRTHCTL_J] Natural family planning methods (also called calendar/rhythm method, cycle beads, basal body temperature).** *Natural family planning methods include behaviors or strategies that women and their partners may use to prevent pregnancy without medication. Some natural family planning methods include the “Calendar method” or the “rhythm method” which mean that couples do not have penile-vaginal sex during the time that a woman is most likely to get pregnant (women are most fertile, or most likely to get pregnant half-way between her menstrual cycle). Women may also use cycle beads (See picture below) to help her keep track of when she is most likely to get pregnant. Every bead represents a day of the month and she should not have sex during the days that are represented by the white beads. Finally, some women using natural family planning methods rely on the basal body temperature method. This method requires women to track her temperature and her cervical fluid every day. There is point in the month during which her temperature and cervical fluid changes and that signals the time that she is most likely to get pregnant and therefore she should avoid having sex.”*
24. Yes
25. No
26. Don’t know
27. Prefer not to answer
28. **[BIRTHCTL_K] Emergency contraception (morning after pill, also known as “Plan B®” or Ella®)**
29. Yes
30. No
31. Don’t know
32. Prefer not to answer
33. **[BIRTHCTL_L] Partner’s vasectomy (also known as male sterilization)**
34. Yes
35. No
36. Don’t know
37. Prefer not to answer
38. **[BIRTHCTL_M] Female sterilizing operation such as tubal sterilization (also called “getting your tubes tied” or having a “tubal ligation”)** *Some women become sterilized by having a small coil inserted inside her fallopian tubes; this method is called “Essure®”.*
39. Yes
40. No
41. Don’t know
42. Prefer not to answer
43. **[BIRTHCTL_N] Any other method not mentioned previously**
44. Yes, *please specify the other method:* _________________________________
45. No
46. Don’t know
47. Prefer not to answer

**[PROGRAMMING NOTE: IF HAVE NEVER USED A BIRTH CONTROL METHOD, PLEASE SKIP TO AFFORDBC.]**

1. **[STOPBC_YN] Did you ever stop using a method because you were not satisfied with it or just didn’t like it?** *Do not count stopping a method for reasons other than dissatisfaction, for example, stopped to get pregnant or because you were not having sex with a male.* [Source: NSFG]
2. Yes
3. No **🡪 GO TO [AFFORDBC]**
4. Prefer not to answer **🡪 GO TO AFFORDBC**
5. **[STOPBC] What method or methods did you stop because you were not satisfied?** *Please check all that apply.* [Source: NSFG]
6. Birth control pills
7. Withdrawal (also called “the pull-out method”)
8. IUD (Mirena®, Paragard®, Skyla®, Liletta®, or Kyleena®)
9. Implant (Implanon® or Nexplanon®)
10. Vaginal ring (Nuvaring® or other)
11. Depo-Provera® (also called “the shot”)
12. Birth control patch (Evra® or other)
13. Male condoms
14. Natural family planning methods (also called calendar/rhythm method, cycle beads, basal body temperature)
15. Barrier methods (diaphragm, sponge, cervical cap, female condom)
16. Prefer not to answer
17. **[AFFORDBC] In the past 12 months, have you wanted a birth control method, but couldn’t afford it?**
18. Yes
19. No
20. Prefer not to answer
21. **[BCTRBL_YN] In the past 12 months, have you delayed or had trouble getting the birth control method you wanted for any reason?** [Source: NSFG]
22. Yes
23. No 🡪 **GO TO [MEDCARE_YN]**
24. Prefer not to answer 🡪 GO TO MEDCARE_YN
25. **[BCTROUBLE] Why did you delay or have trouble getting the birth control method that you wanted?** *Please check all that apply.*
26. I couldn’t afford it.
27. I couldn’t get through to a doctor, clinic, or pharmacy on the telephone.
28. I couldn’t get an appointment with a doctor, clinic, or pharmacy soon enough.
29. The doctor, clinic, or pharmacy wasn’t open when I could get there.
30. Once I got to the doctor, clinic, or pharmacy the wait was too long to see someone.
31. The doctor, clinic, or pharmacy no longer offered the birth control services I need
32. I did not have transportation or a ride to the clinic/pharmacy.
33. I was treated unfairly.

**[BC_UNFAIR] Why were you treated unfairly? I was treated unfairly….** *Please check all that apply.*

- - 1. Because of my race/ethnicity
    2. Because English is not my first language
    3. Because of my sexual orientation
    4. Because of my sexual activity or lifestyle
    5. Some other reason

1. Other, *please specify:* ___________________________________________
2. Prefer not to answer
3. **[MEDCARE_YN] In the past 12 months, have you received any medical care?** *Please include all medical care including urgent care, ER visits, routine doctor visits, and visits with a specialist (e.g., gynecologist, dentist, eye doctor).* [Source: NSFG]
4. Yes
5. No
6. Prefer not to answer
7. **[HEARD_BC] In the past 3 months, have you received or seen any information about birth control methods from any of the following sources?** [Source: Title X survey]

|  | **Yes** | **No** | Don’t know | Prefer not to answer |
| --- | --- | --- | --- | --- |
| 1. A friend or family member | 1 | 2 | 77 | 99 |
| 1. Twitter, Facebook, Instagram, or Snapchat | 1 | 2 | 77 | 99 |
| 1. Other social media, online advertisements, Google, websites, or internet sources | 1 | 2 | 77 | 99 |
| 1. Posters, signs, or billboards | 1 | 2 | 77 | 99 |
| 1. TV or Radio | 1 | 2 | 77 | 99 |
| 1. Ads or campaigns in the community, such as at bars, restaurants, or other local events | 1 | 2 | 77 | 99 |
| 1. Print ads, such as in magazines, newspapers, and brochures | 1 | 2 | 77 | 99 |
| 1. A nurse, doctor, or other healthcare provider | 1 | 2 | 77 | 99 |
| 1. Social worker or community health worker | 1 | 2 | 77 | 99 |
| 1. Other source 🡪 *Please specify any other source(s)*: ­­­­­­­­­­­­­­­­­­­­­­­­­­­­­­­­­­   ______________________________________ | 1 | 2 | 77 | 99 |

**[PROGRAMMING NOTE: IF ANSWERED YES TO ANY ITEMS IN HEARD_BC, PLEASE CONTINUE TO INFOLEARNED, ELSE SKIP TO PAY_BC.]**

1. **[INFOLEARNED] What have you learned from these sources?** *Please check all that apply.*
2. Where you can go to get birth control methods
3. How much different birth control methods cost
4. What types of birth control methods are the most effective at preventing pregnancy
5. Information about a particular birth control method, such as how it is placed or how it works
6. Other information, *Please specify:* ________________________________________
7. Prefer not to answer
8. **[PAY_BC] Do you know how YOU can get any of the following birth control methods for free (by FREE we mean you don’t have to pay anything out-of-pocket)?** [Source: Delaware Household Survey]

| Method | Yes | No | Prefer not to answer |
| --- | --- | --- | --- |
| 1. Male condoms | 1 | 2 | 99 |
| 1. IUD (Mirena®, Paragard®, Skyla®, Liletta®, or Kyleena®) | 1 | 2 | 99 |
| 1. Implant (Nexplanon®) | 1 | 2 | 99 |
| 1. Depo-Provera® (also called “the shot”) | 1 | 2 | 99 |
| 1. Birth control pills | 1 | 2 | 99 |
| 1. NuvaRing® (vaginal birth control ring) | 1 | 2 | 99 |
| 1. Other method(s): please list:_________________ | 1 | 2 | 99 |

1. **[BC_TF] True or False: You can start any method of birth control that you want during one office visit to a clinic.**
   1. True
   2. False
   3. It depends on the type of birth control method
2. Don’t know
3. Prefer not to answer

**[PROGRAMMER NOTE: IF P_VERSION=1, DISPLAY VERSION A (1A, 1B); IF P_VERSION=2, DISPLAY VERSION B (2A, 2B).]**

1. **[ABLIST_1A:] From the following list of health experiences, how many have you personally experienced? You don’t need to say which ones, just how many.** *Enter 0 if you had none of those experiences, 1 if you had any one experience, 2 if you had any two experiences, and 3 if you had all three experiences.* [Source: Ibis Reproductive Health and Texas PEP]

- Ever used or taken any medication for which a prescription is needed

- Ever had a pap smear

- Diagnosed with breast cancer in the past 10 years

|  | *Enter number between 0-3* |
| --- | --- |

1. Don’t Know
2. Prefer not to answer
3. **[ABLIST_2A:] From the following list of health experiences, how many have you personally experienced? You don’t need to say which ones, just how many.** *Enter 0 if you had none of those experiences, 1 if you had any one experience, 2 if you had any two experiences, 3 if you had any three experiences, and 4 if you had all four experiences.* [Source: Ibis Reproductive Health and Texas PEP]

- Ever used or taken any medication for which a prescription is needed

- Ever had a pap smear

- Ever had an abortion (ended a pregnancy on purpose)

- Diagnosed with breast cancer in the past 10 years

|  | *Enter number between 0-4* |
| --- | --- |

1. Don’t Know
2. Prefer not to answer
3. **[ABLIST_1B:] From this next list of health experiences, how many have you personally experienced? You don’t need to say which ones, just how many.** *Enter 0 if you had none of those experiences, 1 if you had any one experience, 2 if you had any two experiences, 3 if you had any three experiences, and 4 if you had all four experiences.* [Source: Ibis Reproductive Health and Texas PEP]

- Ever used a birth control method (such as: pills, an IUD or implant, condoms, or the shot)

- Ever had an abortion (ended a pregnancy on purpose)

- Had an ectopic or tubal pregnancy in the past year

- Ever had your blood pressure measured

|  | *Enter number between 0-4* |
| --- | --- |

1. Don’t Know
2. Prefer not to answer
3. **[ABLIST_2B:] From this next list of health experiences, how many have you personally experienced? You don’t need to say which ones, just how many.** *Enter 0 if you had none of those experiences, 1 if you had any one experience, 2 if you had any two experiences, and 3 if you had all three experiences.* [Source: Ibis Reproductive Health and Texas PEP]

- Ever used a birth control method (such as: pills, an IUD or implant, condoms, or the shot)

- Had an ectopic or tubal pregnancy in the past year

- Ever had your blood pressure measured

|  | *Enter number between 0-3* |
| --- | --- |

1. Don’t Know
2. Prefer not to answer

**In the following section, we’d like to ask your opinions about abortion. Please consider your own thoughts, opinions, and experiences when answering the following questions.**

1. **[ABRT_EASE] Based on what you know or have heard, how easy or difficult is it for a woman to obtain an abortion in your state?** [Source: Guttmacher]
2. Very easy
3. Somewhat easy
4. Neither easy nor difficult
5. Somewhat difficult
6. Very difficult
7. It depends on the situation
8. Don’t know
9. Prefer not to answer
10. **[ABRT_SAFE] Based on what you know or have heard, how safe or dangerous do you think abortion is in your state?** [Source: Guttmacher]
11. Very safe
12. Somewhat safe
13. Neither safe nor dangerous
14. Somewhat dangerous
15. Very dangerous
16. It depends on the situation
17. Don’t know
18. Prefer not to answer
19. **[ABRT_REGRET] Based on what you know or have heard, how likely is it that a woman will regret having an abortion?** [Source: Guttmacher]
20. Very likely
21. Somewhat likely
22. Neither likely nor unlikely
23. Somewhat unlikely
24. Very unlikely
25. It depends on the situation
26. Don’t know
27. Prefer not to answer

**To what extent do you agree or disagree with the following statements:** [Source: Guttmacher]

|  | Strongly agree | Agree | Neither agree nor disagree | Disagree | Strongly disagree | Prefer not to answer |
| --- | --- | --- | --- | --- | --- | --- |
| 1. **[ABRT_AVAIL]** A range of safe, effective, and affordable methods of abortion care should be available to women in their community. | 1 | 2 | 3 | 4 | 5 | 99 |
| 1. **[ABRT_ARREST]** A woman should not fear being arrested or going to jail for having an abortion (terminating a pregnancy). | 1 | 2 | 3 | 4 | 5 | 99 |

1. **[DR_FAVOR]** **How favorably or unfavorably do you view doctors who provide abortion?** [Source: NARAL]
2. Very favorably
3. Somewhat favorably
4. Neither favorably nor unfavorably
5. Somewhat unfavorably
6. Very unfavorably
7. It depends on the situation
8. Prefer not to answer
9. **[ABRT_VIEW] Which comes closest to your own view on abortion?** [Source: NARAL]
10. Having an abortion is acceptable
11. Having an abortion may be acceptable depending on the circumstances
12. Having an abortion is never acceptable
13. Prefer not to answer
14. **[ABRT_IDENT]** **Do you identify as…?**
15. Pro-Choice
16. Pro-Life
17. Neither
18. Both
19. Prefer not to answer

**Now we would like to ask you a few questions about your background.**

1. **[EDUC]** **What is the highest degree or level of school you have completed?**
2. No schooling completed
3. Nursery school
4. Kindergarten
5. Grade 1 through 11, *Specify grade:* _____________________
6. 12^th^ grade – No diploma
7. Regular high school diploma
8. GED or alternative credential
9. Some college credit, but less than 1 year of college credit
10. 1 or more years of college credit, no degree
11. Associate’s degree (for example: AA, AS)
12. Bachelor’s degree (for example: BA, BS)
13. Master’s degree (for example: MA, MS, MEng, MEd, MSW, MBA)
14. Professional degree beyond a bachelor’s degree (for example: MD, DDS, DVM, LLB, JD)
15. Doctorate degree (for example: PhD, EdD)
16. Prefer not to answer
17. **[BORN] Where were you born?** [Source: ACS]
18. United States (including its territories: Puerto Rico, Guam, etc.)
19. Outside of the United States
20. Prefer not to answer
21. **[ETHNICITY] Are you of Hispanic, Latina, or Spanish origin?** [Source: BRFFS]
22. Yes
23. No
24. Prefer not to answer
25. **[RACE] Which of the following best describes your race?** *Please check all that apply*.
26. Black or African American
27. White
28. Asian or Asian American
29. Native American, Alaska Native, or American Indian
30. Native Hawaiian or Pacific Islander
31. Other, *please specify:* ____________________________________
32. Prefer not to answer
33. **[MARITAL] What is your current marital status?** [Source: modified from BRFFS]
34. Now married **🡪 GO TO ORIENTATION**
35. Separated
36. Widowed
37. Divorced
38. Never married
39. Prefer not to answer
40. **[COUPLE] Do you currently live with a romantic partner?**
41. Yes
42. No
43. Prefer not to answer
44. **[ORIENTATION] Which of the following best represents how you think about yourself?** [Source: NSSHB]
45. Lesbian or gay
46. Straight, that is, not lesbian or gay
47. Bisexual
48. Something else
49. Don't Know
50. Prefer not to answer
51. **[DESC_GENDER] How do you describe yourself?** [Source: 2015 U.S. Transgender Survey (USTS)]
52. Female
53. Male
54. Transgender
55. Do not identify as female, male, or transgender
56. Don't know
57. Prefer not to answer
58. **[STUDENT] At any time IN THE LAST 3 MONTHS, have you attended school or college?** *Include only schooling which leads to a high school diploma, or a college, graduate, or professional degree.* [Source: ACS]
59. Yes
60. No
61. Prefer not to answer
62. **[EMPLOY] Are you currently…?** [Source: BRFFS]
63. Employed for wages
64. Self-employed
65. Student
66. Out of work for 1 year or more
67. Out of work for less than 1 year
68. A homemaker
69. Retired
70. Unable to work
71. Prefer not to answer
72. **[INCOME] In studies like this, people are often grouped according to the income shared by an entire household. Thinking about your household’s income from all sources in 2015, what was the approximate income earned by everyone, *before taxes*? Your best guess is fine.** *If you are a student, please include the household income where you currently live even if that includes a dorm room or apartment that you rent only during the school year.*

| $ |
| --- |

1. Don’t know
2. Prefer not to answer

**[INCOME_PROBE] How about if I give you some categories? Would you say your household’s income in 2015 was…**

1. Less than $10,000
2. $10,000 to less than $15,000
3. $15,000 to less than $20,000
4. $20,000 to less than $25,000
5. $25,000 to less than $35,000
6. $35,000 to less than $50,000
7. $50,000 to less than $75,000
8. $75,000 or more
9. Don't Know
10. Prefer not to answer
11. **[INCOME_YN] Now, please consider just yourself. During the past 12 MONTHS, did YOU receive any income from wages, salary, commissions, bonuses, or tips?**
12. Yes
13. No **🡪 GO TO HOURS_WEEK**
14. Prefer not to answer 🡪 GO TO HOURS_WEEK
15. **[INCOME_SELF] What was the total amount of income YOU received for the PAST 12 MONTHS?** *Please report the amount you earned before taxes were taken out. Your best guess is fine. If you are a student, please DO NOT include student loans or grants. You can include stipends.*

| **$** |
| --- |

1. Don’t Know
2. Prefer not to answer
3. **[HOURS_WEEK] During the PAST 12 MONTHS, how many hours did you usually work each WEEK?** [Source: ACS]

_____________

1. Don’t Know
2. Prefer not to answer
3. **[RELIGIOUS] Currently, how important is religion in your daily life? Would you say it is very important, somewhat important, or not important?** [Source: CBS News/New York Times Abortion Poll (July 1989)]
4. Very important
5. Somewhat important
6. Not important
7. Don’t know
8. Prefer not to answer
9. **[RELIG_SERV] About how often do you attend religious services?** [Source: Fog Zone]
10. More than once a week
11. Once a week
12. 2-3 times a month
13. Once a month (about 12 times a year)
14. 3-11 times a year
15. Once or twice a year
16. Never **🡪 GO TO RENTOWN**
17. Prefer not to answer
18. **[RELIG_DESC] Please specify your religious preference (e.g., Catholic, Protestant, Muslim).**

|  |
| --- |

1. Prefer not to answer
2. **[RENTOWN] In the next set of questions, we’d like to ask about your current living situation. Is the**

**house, apartment, or mobile home that you’re living in…** [Source: ACS]

1. Owned by you or someone in this household with a mortgage or loan (*Include home equity loans.)*
2. Owned by you or someone in this household free and clear (without a mortgage or loan)
3. Rented
4. Occupied without payment of rent
5. Don't Know
6. Prefer not to answer
7. **[PPL_HOME] Including you, how many people have been living or staying at this address for more than 2 months?**

|  |
| --- |

1. Don’t know
2. Prefer not to answer
3. **[CHILD_HOME] How many children less than 18 years of age live in your household?** [Source: BRFFS]

|  |
| --- |

1. Don’t know
2. Prefer not to answer
3. **[HOME_1YR] Did you live in this house or apartment 1 year ago?** [Source: ACS]
4. Yes **🡪 GO TO [TUBAL]**
5. No, I lived in a different house or apartment in [STATE]
6. No, I lived in a different house or apartment in another state
7. No, I lived in a different house or apartment outside of the United States
8. Prefer not to answer
9. **[HOME_2MOS] Have you been living or staying at your current address for more than 2 months?** [Source: ACS]
10. Yes
11. No
12. Prefer not to answer

**In this next section, we’d like to learn more about your reproductive health.**

1. **[TUBAL] Have you had a tubal ligation (“tubes tied” or “Essure”) or another operation that makes you unable to get pregnant**? [Source: Modified from NSFG]
2. Yes **🡪 GO TO [PREG_LIFE]**
3. No
4. Prefer not to answer
5. **[INFERTILE] Have you ever been diagnosed as infertile?** [Source: Fog Zone]
6. Yes **🡪 THANK YOU. THESE ARE ALL OF THE QUESTIONS WE HAVE FOR YOU NOW.**
7. No
8. Prefer not to answer
9. **[CURR_PREG] Are you currently pregnant?** [Source: NSFG]
10. Yes **🡪 GO TO [FEEL_CHILD]**
11. No
12. Don’t Know
13. Prefer not to answer
14. **[TRYING_PREG] Are you currently trying to get pregnant?** [Source: NSFG]
15. Yes **🡪 GO TO FEEL_CHILD**
16. No
17. Prefer not to answer
18. **[PRSNL_DR] Do you have at least one person or place you think of as your personal doctor or health care provider?** [Source: modified from BRFSS]
19. Yes
20. No
21. Prefer not to answer
22. **[LASTDRVISIT] About how long has it been since you last visited a doctor or other healthcare provider for a routine checkup?** *A routine checkup is a general physical exam, not an exam for a specific injury, illness, or condition.* [Source: modified from BRFSS]
23. Within the past year (anytime less than 12 months ago)
24. Within the past 2 years (1 year but less than 2 years ago)
25. Within the past 5 years (2 years but less than 5 years ago)
26. 5 or more years ago
27. Never
28. Don’t know
29. Prefer not to answer
30. **(RECD_DR] In the past 12 months, have you received from a doctor or other healthcare provider:** [Source: modified from NSFG]

|  | Yes | No | Don’t know | Prefer not to answer |
| --- | --- | --- | --- | --- |
| a) A method of birth control or a prescription for a birth control method | 1 | 2 | 77 | 99 |
| b) A check-up or medical test related to using a birth control method | 1 | 2 | 77 | 99 |
| c) Counseling or information about birth control | 1 | 2 | 77 | 99 |
| d) A question about whether you want to become pregnant in the next year | 1 | 2 | 77 | 99 |
| e) A pregnancy test | 1 | 2 | 77 | 99 |
| f) A yearly check-up (annual women’s visit) | 1 | 2 | 77 | 99 |

1. **[HLTH_INS] Are you currently covered by any of the following types of health insurance?**

|  | Yes | No | Don’t know | Prefer not to answer |
| --- | --- | --- | --- | --- |
| a) Insurance through current or former employer or union (by you or another family member). This would include COBRA coverage | 1 | 2 | 77 | 99 |
| b) Insurance purchased directly from an insurance company (by you or another family member). This would include coverage purchased through an exchange or marketplace, such as [STATE SPECIFIC OPTION] | 1 | 2 | 77 | 99 |
| c) Medicare, for people 65 and older or people with certain disabilities | 1 | 2 | 77 | 99 |
| d) Medicaid, Medical Assistance (MA), the Children’s Health Insurance Program (CHIP) or any kind of state or government-sponsored assistance plan based on income or a disability. You may know this type of coverage as [STATE SPECIFIC OPTION]. | 1 | 2 | 77 | 99 |
| f) TRICARE or other military health care, including VA health care | 1 | 2 | 77 | 99 |
| g) Indian Health Service | 1 | 2 | 77 | 99 |
| i) Any other type of health insurance or health coverage plan  *Please specify any other plans:* __________________ | 1 | 2 | 77 | 99 |

1. **[CURRUSE_YN] Are you currently using any method or methods of birth control?** [Source: modified from NSFG]
2. Yes **🡪 GO TO [BC_CURR]**
3. No 🡪 **GO TO [NOCONTRA]**
4. Prefer not to answer 🡪 GO TO BC_CURR
5. **[NOCONTRA] What are your reasons for not using any method of birth control?** *Please check all that apply.* [Source: modified from NSFG]
6. I’m not currently having sex with a male.
7. I just don’t think about it.
8. I don’t mind if I get pregnant.
9. I want to get pregnant.
10. I don’t want to use a birth control method.
11. My partner doesn’t want to use a birth control method.
12. I don’t think I can get pregnant.
13. I stopped using birth control methods due to side effects (such as bleeding, cramping, mood swings).
14. I’m currently breastfeeding.
15. I had a problem getting birth control when I needed it.
16. I couldn’t pay for birth control.
17. I think my partner is sterile and cannot impregnate me.
18. I think that I might be infertile or it might be impossible for me to get pregnant.
19. Religious reasons.
20. I recently stopped using my birth control method and haven’t started it again.
21. I just had a baby.
22. Other, *please specify:* __________________________________
23. Prefer not to answer

**[PROGRAMMING NOTE: IF ANSWERED NOCONTRA, PLEASE GO TO FREQ_SEX.]**

1. **What kind(s) of birth control method(s) are you currently using?** *Please check all that apply.* [Source: modified from BRFSS]
2. **[BC_CURR_A] Withdrawal (also called “the pull-out method” when the man “pulls out” during penile-vaginal sex)**
3. Yes

**[LASTSEX_A] Were you using this method the last time you had sex with a male?**

- 1. Yes
  2. No

1. Don’t know
2. Prefer not to answer
3. No
4. Prefer not to answer
5. **[BC_CURR_B] Birth control pills** *Please mark YES even if you are taking birth control pills for reasons other than to prevent pregnancy.*
6. Yes

**[LASTSEX_B] Were you using this method the last time you had sex with a male?**

- 1. Yes
  2. No
  3. Don’t know
  4. Prefer not to answer

1. No
2. Prefer not to answer
3. **[BC_CURR_C] Birth control patch (Evra® or other)**
4. Yes

**[LASTSEX_C] Were you using this method the last time you had sex with a male?**

1. Yes
2. No
3. Don’t know
4. Prefer not to answer
5. No
6. Prefer not to answer
7. **[BC_CURR_D] Vaginal ring (Nuvaring® or other)**
8. Yes

**[LASTSEX_D] Were you using this method the last time you had sex with a male?**

- 1. Yes
  2. No

1. Don’t know
2. Prefer not to answer
3. No
4. Prefer not to answer
5. **[BC_CURR_E] Depo-Provera® (also called “the shot”)**
6. Yes

**[LASTSEX_E] Were you using this method the last time you had sex with a male?**

1. Yes
2. No
3. Don’t know
4. Prefer not to answer
5. No
6. Prefer not to answer
7. **[BC_CURR_F] IUD (Mirena®, Paragard®, Skyla®, Liletta®, or Kyleena®)**
8. Yes

**[CURR_IUD] What IUD are you currently using?**

1. Mirena**®**
2. Paragard**®**
3. Skyla**®**
4. Liletta**®**
5. Kyleena**®**
6. Prefer not to answer

**[LASTSEX_F] Were you using this method the last time you had sex with a male?**

1. Yes
2. No
3. Don’t know
4. Prefer not to answer
5. No
6. Prefer not to answer
7. **[BC_CURR_G] Implant (Implanon® or Nexplanon®)**
8. Yes

**[LASTSEX_G] Were you using this method the last time you had sex with a male?**

1. Yes
2. No
3. Don’t know
4. Prefer not to answer
5. No
6. Prefer not to answer
7. **[BC_CURR_H] Male condoms**
8. Yes

**[LASTSEX_H] Were you using this method the last time you had sex with a male?**

1. Yes
2. No
3. Don’t know
4. Prefer not to answer
5. No
6. Prefer not to answer
7. **[BC_CURR_I] Other barrier methods (diaphragm, sponge, cervical cap, female condom)**
8. Yes

**[LASTSEX_I] Were you using this method the last time you had sex with a male?**

1. Yes
2. No
3. Don’t know
4. Prefer not to answer
5. No
6. Prefer not to answer
7. **[BC_CURR_J] Natural family planning methods (also called calendar/rhythm method, cycle beads, basal body temperature).**
8. Yes

**[LASTSEX_J] Were you using this method the last time you had sex with a male?**

1. Yes
2. No
3. Don’t know
4. Prefer not to answer
5. No
6. Prefer not to answer
7. **[BC_CURR_K] Emergency contraception (morning after pill, also known as “Plan B®” or Ella®)**
8. Yes

**[LASTSEX_K] Did you use this method after the last time you had sex with a male?**

1. Yes
2. No
3. Don’t know
4. Prefer not to answer
5. No
6. Prefer not to answer
7. **[BC_CURR_L] Partner’s vasectomy (also known as male sterilization)**
8. Yes

**[LASTSEX_L] Were you using this method the last time you had sex with a male?**

1. Yes
2. No
3. Don’t know
4. Prefer not to answer
5. No
6. Prefer not to answer
7. **[BC_CURR_M] Other method**
8. Yes, *please specify the other method: _____________________________________*

**[LASTSEX_L] Were you using this method the last time you had sex with a male?**

1. Yes
2. No
3. Don’t know
4. Prefer not to answer
5. No
6. Don’t know
7. Prefer not to answer
8. **[EMERGENCY] In the past 12 months, have you used any of the following forms of emergency contraception (contraception that you used after you had sex)?** [Source: NSFG]

|  | Yes | No | Prefer not to answer |
| --- | --- | --- | --- |
| 1. I used Plan B One-Step® (or a generic like Next Choice® or My Way®) and I got it over-the-counter. | 1 | 2 | 99 |
| 1. I used Plan B One-Step® (or a generic like Next Choice® or My Way®) and I got it with a prescription. | 1 | 2 | 99 |
| 1. I used Ella® (prescription-only emergency contraception). | 1 | 2 | 99 |
| 1. I had an IUD placed for emergency contraception (after unprotected sex). | 1 | 2 | 99 |

1. **[FREQ_SEX] Thinking about the past 3 months, about how often did you have sex with a male?** *By sex, we mean a penis was inserted in your vagina*.
2. About once a week or more
3. A few times a month
4. About once a month
5. Less than once a month
6. I did not have sex with a male in the past 3 months
7. Don’t Know
8. Prefer not to answer
9. **[HI_PAY] Do you use your health insurance to help pay for your birth control method?**
10. Yes
11. No, I chose not to use my health insurance to pay for my birth control even though my insurance covers it.
12. No, my health insurance plan does not cover my birth control method.
13. N/A, I’m not using a prescription method of birth control.
14. N/A, I don’t have health insurance.
15. Prefer not to answer
16. **[BC_SATIS] How satisfied are you with your current birth control method?** *If using more than one method, please report on your satisfaction with the method you use most often.*
17. Very satisfied
18. Somewhat satisfied
19. Neither satisfied nor dissatisfied
20. Somewhat dissatisfied
21. Very dissatisfied
22. Prefer not to answer
23. **[BC_CONFID] How confident are you that you have been using your method of birth control correctly for the past 3 months?**
24. Completely confident
25. Somewhat confident
26. Neither confident nor not confident
27. Somewhat not confident
28. Not at all confident
29. Prefer not to answer
30. **[BC_SWITCH] Switching your current birth control method and using another method of birth control in the next 3 months is:**
31. Very likely
32. Somewhat likely
33. Neither likely nor unlikely
34. Somewhat unlikely
35. Very unlikely
36. Prefer not to Answer
37. **[BC_CONTROL] Your use of your birth control method in the past 3 months was:**
38. Completely under my control
39. Somewhat under my control
40. Neither under my control nor out of my control
41. Somewhat out of my control
42. Not at all under my control
43. Prefer not to answer
44. **[BC_FRIENDS] How many of your friends will use a birth control method in the next 3 months?**
45. Almost all of them
46. Most of them
47. About half of them
48. Less than half of them
49. Almost none of them
50. Don't know
51. Prefer not to answer
52. **[FEEL_CHILD] How do you feel about having a child now or sometime in the future?** [Source: BRFFS]
53. I don’t want to have one
54. I do want to have one, less than 12 months from now
55. I do want to have one, between 12 months and less than 2 years from now
56. I do want to have one, between 2 years to less than 5 years from now
57. I do want to have one, five or more years from now
58. I do want to have one, but I’m not sure when
59. I don’t know
60. Prefer not to answer
61. **[PREGFEELNS] Please think about how you CURRENTLY feel about the following statements:** [Source: Fog Zone]

|  | Strongly agree | Agree | Neither agree nor disagree | Disagree | Strongly disagree | Not Applicable | Prefer not to answer |
| --- | --- | --- | --- | --- | --- | --- | --- |
| 1. It doesn’t matter whether you use birth control or not, when it is your time to get pregnant, it will happen | 1 | 2 | 3 | 4 | 5 | 6 | 99 |
| 1. Having a baby in the next year could mess up my life | 1 | 2 | 3 | 4 | 5 | 6 | 99 |
| 1. Getting pregnant would bring me and my partner closer together | 1 | 2 | 3 | 4 | 5 | 6 | 99 |
| 1. Even though I’m not trying to get pregnant, I would be happy if it happened | 1 | 2 | 3 | 4 | 5 | 6 | 99 |
| 1. If I got pregnant in the next year, I would consider having an abortion | 1 | 2 | 3 | 4 | 5 | 6 | 99 |
| 1. Getting pregnant would make my partner happy | 1 | 2 | 3 | 4 | 5 | 6 | 99 |
| 1. Every pregnancy is a blessing | 1 | 2 | 3 | 4 | 5 | 6 | 99 |
| 1. If I got pregnant in the next year, I would be excited | 1 | 2 | 3 | 4 | 5 | 6 | 99 |

1. **[PREG_AVOID] How important is it to you to AVOID becoming pregnant now?** [Source: Guttmacher]
2. Very important
3. Somewhat important
4. Neither important nor unimportant
5. Somewhat unimportant
6. Not at all important
7. Prefer not to answer
8. **[PREG_LIFE] In your lifetime, have you ever been pregnant?** *Please include pregnancies that ended in miscarriage or abortion, in addition to births*. [Source: NSFG]
9. Yes
10. No **🡪 THANK YOU. THESE ARE ALL OF THE QUESTIONS WE HAVE FOR YOU NOW.**
11. Don’t know **🡪 THANK YOU. THESE ARE ALL OF THE QUESTIONS WE HAVE FOR YOU NOW.**
12. Prefer not to answer **🡪 THANK YOU. THESE ARE ALL OF THE QUESTIONS WE HAVE FOR YOU NOW.**
13. **[PREG_NUM] In your lifetime, how many times have you been pregnant (whether those pregnancies resulted in babies born alive, stillbirth, abortion, miscarriage, or ectopic or tubal pregnancy)?** *If you are currently pregnant, please count your current pregnancy.* [Source: NSFG]

__________

1. Don't Know
2. Prefer not to answer
3. **[PREG_UNPLAN] Have you ever gotten pregnant when you were not planning or wanting to become pregnant?** *Please include pregnancies that ended in miscarriage or abortion, in addition to births.* [Source: Guttmacher]
4. Yes
5. No
6. Don't Know
7. Prefer not to answer
8. **[ALIVE_NUM] How many babies have you had that were born alive?** [Source: NSFG]

__________

1. Prefer not to answer
2. **[MISCAR_NUM] How many times have you had a miscarriage (lost a pregnancy by accident)?** [Source: NSFG]

__________

1. Don't Know
2. Prefer not to answer
3. **[GIVENBIRTH] Have you given birth in the past 12 months?** [Source: modified from NSFG]
4. Yes
5. No **🡪 THANK YOU. THESE ARE ALL OF THE QUESTIONS WE HAVE FOR YOU NOW.**
6. Prefer not to answer **🡪 THANK YOU. THESE ARE ALL OF THE QUESTIONS WE HAVE FOR YOU NOW.**
7. **[BABY_FEEL] Thinking back to just before you got pregnant with your new baby, how did you feel about becoming pregnant?** [Source: PRAMS]
8. I wanted to be pregnant later
9. I wanted to be pregnant sooner
10. I wanted to be pregnant then
11. I didn’t want to be pregnant then or at any time in the future
12. I wasn’t sure what I wanted
13. Prefer not to answer
14. **[BABY_TRY] When you got pregnant with your new baby, were you trying to get pregnant?** [Source: PRAMS]
15. Yes 🡪 **GO TO BABY_HUSBAND**
16. No
17. Prefer not to answer
18. **[BABY_PRVNT] When you got pregnant with your new baby, were you or your partner doing anything to keep from getting pregnant?** [Source: PRAMS]
19. Yes 🡪 **GO TO BABY_HUSBAND**
20. No
21. Prefer not to answer **🡪 GO TO BABY_HUSBAND**
22. **[BABY_NOMIND] What were the reasons you or your partner were not doing anything to keep from getting pregnant?** *Please check all that apply.*
23. I didn’t mind if I got pregnant.
24. I thought I could not get pregnant at that time.
25. I had side effects from the birth control method I was using.
26. I had problems getting birth control when I needed it.
27. I thought my partner or I was sterile (could not get pregnant at all).
28. My partner didn’t want to use anything.
29. I forgot to use a birth control method.
30. Other reason, *please specify:* ­­­­­­­­­­­­­­­__________________________________________
31. Prefer not to answer
32. **[BABY_HUSBAND] During the 12 months before your new baby was born, did your partner say he/she didn’t want you to be pregnant?** [Source: PRAMS]
33. Yes
34. No
35. Prefer not to answer
36. **[BABY_HAPPY] How did you feel when you found out you were pregnant with your new baby? Were you…** [Source: PRAMS]
37. Very unhappy to be pregnant
38. Unhappy to be pregnant
39. Neither happy nor unhappy
40. Happy to be pregnant
41. Very happy to be pregnant
42. Prefer not to answer
43. **[BABY_HI] During the year in which you were pregnant with and gave birth to your new baby, were you covered by health insurance from any of the following?** [Source: PRAMS]
44. Insurance through current or former employer or union (by you or another family member)
45. Insurance purchased directly from an insurance company (by you or another family member)
46. Medicare (for people 65 and older or people with certain disabilities)
47. Medicaid, Medical Assistance (MA), Children’s Health Insurance Program (CHIP) or any kind of state or government-sponsored assistance plan based on income or a disability
48. TRICARE or other military health care, including VA health care
49. Indian Health Service
50. Any other type of health insurance or health coverage plan, *please specify:* _____­­___________
51. I did not have health insurance during my pregnancy
52. Don't Know
53. Prefer not to answer
54. **[PRENATALCARE] Did you have any prenatal care during this most recent pregnancy?** *Prenatal care is given by a healthcare provider and includes a physical exam, weight checks, and providing a urine sample. Depending on the stage of the pregnancy, healthcare providers may also do blood tests and imaging tests, such as ultrasound exams. These visits also include discussions about the mother’s health, the infant’s health, and any questions about the pregnancy.* [Source: PRAMS]
55. Yes
56. No 🡪 **GO TO PRETERM**
57. Prefer not to answer
58. **[PRENATALVST] During any of your prenatal care visits, did a doctor, nurse, or other healthcare provider talk with you about your plans and timing for having another baby?**
59. Yes
60. No
61. Don’t know
62. Prefer not to answer
63. **[PREG_AGAIN] During any of your prenatal care visits, did a doctor, nurse, or other healthcare provider ask you if you want to become pregnant again within the next year?**
64. Yes
65. No
66. Don’t know
67. Prefer not to answer
68. **[PRETERM] A preterm delivery is one that occurs at 36 weeks or earlier in pregnancy. As far as you know, did you have a preterm delivery?** [Source: NSFG]
69. Yes
70. No
71. Prefer not to answer
72. **[BABY_WGT] When your baby was born, did she/he weigh 5 ½ pounds or more?** [Source: NSFG]
73. My baby weighed 5 1/2 pounds or more
74. My baby weighed less than 5 1/2 pounds
75. Prefer not to answer
76. **[PP_BC] Either before or after you gave birth, did a doctor, nurse, or other healthcare provider talk with you about postpartum contraception (or birth control methods that you could start using once your baby was born)?**
77. Yes
78. No
79. Don’t know
80. Prefer not to answer
81. **[PP_BC_6WKS] Did you start a new birth control method within 6 weeks after you gave birth?**
82. Yes
83. No **🡪 THANK YOU. THESE ARE ALL OF THE QUESTIONS WE HAVE FOR YOU NOW.**
84. Prefer not to answer **🡪 THANK YOU. THESE ARE ALL OF THE QUESTIONS WE HAVE FOR YOU NOW.**
85. **[PP_BC_WHICH] What birth control method did you start after giving birth?** *Please check all that apply.*
86. Birth control pills
87. Withdrawal (also called “the pull-out method”)
88. IUD (Mirena®, Paragard®, Skyla®, Liletta®, or Kyleena**®**)

**[PP_IUDUSE] What IUD did you use?**

1. Liletta® (hormonal IUD used for up to 3 years)
2. Mirena® (hormonal IUD used for up to 5 years)
3. Skyla® (hormonal IUD used for up to 3 years)
4. Paragard® (non-hormonal IUD used for up to 10 years, also called the “copper IUD”)
5. Kyleena**®** (hormonal IUD used for up to 5 years)

**[PP_IUDTRBL] Have you had any trouble with the IUD since it was inserted? For example, did it ever need to be re-inserted?**

- 1. Yes 🡪 *Please specify difficulty:* __________________________________
  2. No

1. Implant (Implanon® or Nexplanon®)
2. Vaginal ring (Nuvaring® or other)
3. Depo-Provera® (also called “the shot”)
4. Birth control patch (Evra® or other)
5. Male condoms
6. Natural family planning methods (also called calendar/rhythm method, cycle beads, basal body temperature)
7. Barrier methods (diaphragm, sponge, cervical cap, female condom)
8. Other method, *please specify: _________________________________________*
9. I did not use any method of birth control **🡪 GO TO END**
10. Prefer not to answer

1. **[PP_GETBC] Where did you receive this birth control method?**
2. Before I left the hospital after giving birth
3. At my six-week check-up appointment with my OB/GYN who delivered my baby
4. At my six-week check-up appointment at a family planning clinic
5. Other, *please specify: __________________________________*
6. Prefer not to answer
